# Proposal of a New System for Essential Oil Classification Based on Low-Cost Gas Sensor and Machine Learning Techniques

**DOI:** 10.3390/s23135812

**Published:** 2023-06-22

**Authors:** Sandra Viciano-Tudela, Lorena Parra, Paula Navarro-Garcia, Sandra Sendra, Jaime Lloret

**Affiliations:** Instituto de Investigación para la Gestión Integrada de Zonas Costeras, Universitat Politècnica de València, C/Paranimf, 1, 46730 Gandia, Spain; svictud@upv.es (S.V.-T.); paunagar@alumni.upv.es (P.N.-G.); sansenco@upv.es (S.S.); jlloret@dcom.upv.es (J.L.)

**Keywords:** adulteration, eNose, MQ sensors, classification algorithms, k-nearest neighbours, artificial neural network, discriminant analysis, naive Bayes classifier, support vector machine, multiclass classification

## Abstract

Essential oils are valuable in various industries, but their easy adulteration can cause adverse health effects. Electronic nasal sensors offer a solution for adulteration detection. This article proposes a new system for characterising essential oils based on low-cost sensor networks and machine learning techniques. The sensors used belong to the MQ family (MQ-2, MQ-3, MQ-4, MQ-5, MQ-6, MQ-7, and MQ-8). Six essential oils were used, including *Cistus ladanifer*, *Pinus pinaster*, and *Cistus ladanifer* oil adulterated with *Pinus pinaster*, *Melaleuca alternifolia*, tea tree, and red fruits. A total of up to 7100 measurements were included, with more than 118 h of measurements of 33 different parameters. These data were used to train and compare five machine learning algorithms: discriminant analysis, support vector machine, k-nearest neighbours, neural network, and naive Bayesian when the data were used individually or when hourly mean values were included. To evaluate the performance of the included machine learning algorithms, accuracy, precision, recall, and F1-score were considered. The study found that using k-nearest neighbours, accuracy, recall, F1-score, and precision values were 1, 0.99, 0.99, and 1, respectively. The accuracy reached 100% with k-nearest neighbours using only 2 parameters for averaged data or 15 parameters for individual data.

## 1. Introduction

Essential oils are the volatile liquid fraction which contains the substances responsible for the scent of plants. These are important in cosmetics (perfumes and flavourings), food (condiments and flavouring), and pharmaceutical (flavouring) industries [1]. Essential oils in plants can act as an attractant of pollinators, repel phytophagous, have allelopathic or antibacterial activity, and contribute to maintaining the water level of the plant [2]. They can be extracted from plant samples by different methods: hydrodistillation, steam distillation, hydrodiffusion, solvent extraction, and supercritical fluid extraction [3].

Essential oils are expensive products which can be easily adulterated. When an oil sample is adulterated, it means that a natural or synthetic component has been added during the production process. Therefore, the original oil is no longer pure and has lower quality [4]. Adulteration can be due to different factors: the addition of synthetic material, volatile compounds extracted from other natural resources, the substitution of compounds that were extracted from other plants, and the addition of vegetable oil to increase the weight of the essential oil [5]. The consequences of using adulterated essential oils are the following: (i) modification of the synergy and physiological activities of the oil; (ii) reduction of therapeutic benefits; (iii) increased risk of adverse reactions; and (iv) introduction of toxic compounds into the body [6].

The existing methods to characterise essential oils are costly and highly time consuming. Essential oils are safe except in overdosage, wrong application route (orally, inhaled, or on the skin), and for hypersensitive people. Depending on the essential oil, it must be used in a certain way. If they are not used correctly, there may be side effects such as skin sensibilisation, genotoxicity, neurotoxicity, and reproductive toxicity [7].

The most widely used technique for the characterisation of essential oils is gas chromatography—mass spectrometry (GC/MS). Additional techniques used are liquid chromatography coupled with mass spectrometry (LC-MS) or using other spectroscopic methods, such as infrared spectroscopy or nuclear magnetic resonance spectroscopy (NMR) [8]. Nevertheless, these are chemical techniques which require much time, money, specialised equipment, and trained personnel. The results obtained can be difficult to interpret by people who are not experts in the field and can present the following drawbacks: baseline drift, spectral background, homoscedastic and heteroscedastic noise, peak shape deformation (non-Gaussian peaks), low signal/noise ratio, and coelution [9].

A cost-effective solution that facilitates the characterisation of essential oils is the use of electronic noses (eNoses) as sensors [10]. Unlike the methods mentioned above, these can give a real-time or near-real-time response if they are implemented in a portable way [11]. An eNose is a device which comprises an array of electronic chemical sensors with partial specificity and an appropriate pattern-recognition system capable of recognising simple or complex odours [12]. These devices allow us to obtain information on the chemical and physical nature of substances. Their applications are being developed in new research areas, such as evaluating volatile emissions, homeland safety, environmental protection, biomedical diagnostics, personnel safety, and product development research [13]. In addition, in the food sector, its use has increased for multiple applications such as monitoring fruit quality [14] and classifying grape crops [15] and vegetables [16]. eNoses have also been implemented for the detection of volatile compounds from fertilisers [17] as well as volatile compounds from rapeseed oil pressing [18]. Regarding its use to characterise essential oils, examples of eNoses used for oil detection have been found [19,20,21].

The use of eNoses is strongly related to applying classification techniques such as multivariate data analysis, cluster, and machine learning (ML) [22]. Among the classification techniques, ML is one of the most recent and common methods, highlighting the use of support vector machine (SVM), k-nearest neighbours (KNN), and neural networks (NN) as the most used ones [23]. There are plenty of studies in which eNoses have been used to identify or classify food odours using ML algorithms, including foods such as strawberries, oranges, beer, wine, coffee, cheese, ham, and pears among others [24]. However, according to a recent survey, the combined use of eNoses with ML in identifying oil is limited to one example [24]. In [19] and [21], authors used ML for essential oil classification. Nonetheless, in both cases, just one algorithm was used: SVM in [19] and NN in [21].

Examples of eNoses and ML used to characterise oil can be found in [25,26]. The examples include using NN to determine the oil rancidity and aroma profiles of olive oil [25] and identifying monovarietal olive oils with NN [26]. ML is also used to classify data from Raman spectroscopy to determine the quality of peanut oil [27]. Nevertheless, the application of ML and eNoses in determining essential oils’ origin is still limited to [19,21]. In the study case in which eNoses were used to determine the origin and adulteration of essential oils [20], no comparison of the performance of ML was conducted. Thus, in this study, we aim to compare which ML algorithm offered better performance in determining the origin of different essential oils and compare different data processing techniques as an alternative to the traditional GC/MS method, which is costly, highly time-consuming, and requires specialised personnel.

The aim of this paper is to evaluate the performance of a previously developed eNose for essential oil with a wider variety of samples. New data are gathered from additional sorts of essential oils used in different applications and from different sources. We included five essential oils in our tests. Three of them come mainly from the leaves and branches of the plant, such as the *Cistus ladanifer*, *Pinus pinaster*, and *Melaleuca alternifolia*. The other two essential oils that were used were obtained from the fruits of the *Citrus limonun* and red fruits. New classification techniques were evaluated to complement the previously published experiments, and larger datasets with and without pretreatment were tested. The total generated data include up to 7000 registered values and more than 118 h of data for each of the seven sensors used. Considering that each sensor can measure up to five compounds on average, the total registered values reach 234.762. The classification techniques used in this study include discriminant analysis (DA), SVM, naive Bayesian (NB), NN, and KNN algorithms. The main novelties of this paper include:

- Proposal of a new system for essential oil characterisation based on a low-cost sensor network and ML techniques;
- Comparison of results includes a larger dataset of essential value data, including five sorts of essential oils and an adulterated sample;
- Evaluate the suitability of using individual data or the mean data for classification;
- Assess the best classification algorithm for the obtained data;
- Calculate metrics, precision, accuracy, recall, and F1-score to allow fair comparison of results with existing, ongoing, and future studies;
- Inclusion of the sensor in a sensor network allowing cloud computing.

The contribution of this paper is to provide a low-cost system for data classification in the laboratory integrated into a wireless network using a local database and cloud computing. A comparison of the performance of ML algorithms in the classification of essential oils based on data from a low-cost eNose focusing on the different data treatment options was conducted to determine the best configuration of the proposed system. As far as we are concerned, no similar system has been provided, including their low cost and capability to operate autonomously and wirelessly and classify essential oils.

The rest of the paper is structured as follows; Section 2 outlines the related work. The test bench is fully described in Section 3, including the sensor used, the data gathering methodology, and data processing. Section 4 analyses the obtained results in terms of the performance of different conducted classifications when different data and classification algorithms are used. The obtained results are discussed in Section 5, in which the most relevant findings and main limitations of the present work are highlighted. Finally, the conclusion and future work are summarised in Section 6.

## 2. Related Work

This section summarises different studies carried out through the development of eNoses. Many studies use the MQ family of sensors with microcontrollers, such as Arduino. These sensors, as shown below, have a wide variety of applications that allow the detection of gases and volatile compounds in many fields. This section shows, on the one hand, both the application of these systems in the alimentation industry and the detection of adulteration in different materials. On the other hand, the application in other areas is shown, such as industry, contamination detection, and bacteria detection.

### 2.1. Sensors of the MQ for Detection of Adulteration in Essential Oils and Applications in Food

Since, in many cases, the pure raw materials are highly priced, adulteration is common. There are products in which the possibility of adulteration is carefully controlled to avoid fraud. There are studies carried out that focus on detecting this type of illegal activity. In addition, using gas sensors has made it possible to establish the quality of different foods.

Karami et al., in 2020 [28], selected a set of gas sensors, MQ-3, MQ-9, MQ-135, MQ-136, TGS-813, TGS-822, TGS-2602, and TGS-2620, to develop an eNose. With this system, they detected adulterated edible oils. Regarding the classification methods, cluster analysis (CA), principal components analysis (PCA), linear discriminant analysis (LDA), NN, and others were evaluated. They achieved a mean classification accuracy of 97.3% with NN compared to 88% with LDA. In 2021, Rasekh et al. [21] developed an eNose based on metal oxide semiconductor sensors. With this system, they detected the adulteration of plant products through aroma through counterfeit essential oils. To do this, the authors used the MAU-9 electronic sensory methods. These methods detected the authenticity and quality of the plant product by using the volatile compounds of edible fruits and herbs. An NN was used for data classification. In addition, in terms of statistical analysis, the main regression method and partial least squares were included. The results showed that the NN classified the data obtained in 100% of the cases when applied to two groups. Meanwhile, 98.9% of the cases were correctly classified for the six groups. They also conclude that using all the sensors is unnecessary. That same year, Yavuzer [29] developed an eNose system using the MQ-3, MQ-4, MQ-5, MQ-8, MQ-9, and MQ-135 sensors. The proposed system established the deterioration of sea bream, trout, and sea bass through odour changes. The data obtained using the sensors were compared with microbiological data. The authors established that by using an Arduino microprocessor and the eNose, it was possible to detect the quality of 10 g of fish. As for the price of the box, it is $20.

Wakhid et al. [30], in 2022, evaluated an eNose system based on MQ sensors to detect adulteration of beef meat with pork meat. For this, three chambers of different sizes were tested. The authors studied the effect of the concentration of gases. Their results indicated that the chamber with the highest gas concentration establishes better results with greater precision, having a precision of 95.71% for a 50 mL chamber. The statistical parameters used were kurtosis and asymmetry. The highest classification performance was obtained with the SVM method. Finally, in 2023, Viciano-Tudela et al. [20] developed a gas detection system to detect the adulteration of essential gases from *Cistus ladanifer* with *Pinus pinaster*. Therefore, they developed a gas chamber that allowed the emitted volatile compounds to be concentrated. The achieved results enabled a reduction in the studied parameters and correctly classified 100% of the oils studied using only two MQ sensors (MQ-3 and MQ-8). The statistical analysis carried out was the NN. In addition, the response of the gas sensors over time was evaluated.

### 2.2. Other Applications of MQ Family Sensors

The MQ family of gas sensors are present in many fields. Below, a selection of studies in which electronic noses have been developed for gas detection is outlined.

Wonohardjo and Kusuma [31], in 2019, used the MQ-7 sensor to detect CO pollution. In addition, they used Google Maps to map the areas. The final system comprised the MQ-7 sensor, a display module, an Arduino board, and a web server. With all this, the results showed that the system could provide information about the level of contamination of a place in real time. In the same year, Salinas Alvarez et al. [32] implemented MQ sensors for a novel use. They used the TGS-826, MQ-3, MQ-135, and MQ-138 sensors to detect the presence of bacteria in ulcers. The developed system was validated by measuring the gases at different distances and concentrations. Among the gases detected were ammonia, CO_2_, alcohol, and acetone. They concluded that the agar plate solid cultures presented more significant gas emissions than those carried out in liquid culture. Moreover, they found that the system could detect a more significant number of gases in six strains of *Pseudomonas aeruginosa*. Finally, that same year, Subandri and Sarno [33] presented a study to reduce the use of sensors in eNoses. They used the KNN algorithm. Of the initial ten sensors, they eventually used four for the characterisation of banana samples. The results showed that the precision of the final system was 78%. In this way, it was shown that minimising the number of sensors used is feasible.

In 2020, Fakra et al. [34] proposed a system for characterising combustion gases in isolated areas, considering that in the energy sector, their concentrations are lower. They adopted MQ-4 and MQ-8 sensors using a capsule to measure CH_4_ and H_2_. They used three different classification techniques. In the first method, a closed, airtight chamber was implemented. In the second, the gas was injected directly into the sensor in an open environment. Finally, the gas was injected directly into the sensor using a closed capsule in the third method. The results showed differences among the used techniques. The first technique presented the best repeatability; the standard deviation was 13.88% and 5.1% for CH_4_ and H_2_, respectively. Regarding linearity, the second method presented better values. Nevertheless, it showed poor repeatability. The third technique presented the best R^2^ values of 0.9973 and 0.9472 for CH_4_ and H_2_, respectively.

In 2021, Abdulrazzak et al. [35], using a drone, detected the presence of H_2_S gas in a refinery in Iraq. The drone was composed of a GPS for its location and an MQ sensor to detect the presence of the monitored gas. The drone was programmed using an Arduino system. The results showed that the detected values were within the allowed range from 1058 to 5034. In that same year, Sanger et al. [36] developed a system composed of an Arduino Uno, NodeMCU ESP8266, and gas sensors MQ-136, MQ-137, and TGS-2611 for the detection of toxic gases in garbage. Among the gases detected were CH_4_, hydrogen sulfide, and ammonia. The results obtained by comparing clean air with polluted air were 0.0904 ppm and 44.696 ppm for the MQ-137 sensor, 0.0624 ppm and 9.1884 ppm for the MQ-136 sensor, and 8.6236 ppm and 8128 ppm for the TGS-Sensor 2611.

Finally, in 2023, Kiki et al. [37] implemented an eNose system based on metal oxide semiconductor sensors to detect the fall armyworm plague, which destroys crops and considerably affects the agricultural sector. Therefore, they studied the sensors that showed the most significant response to the volatile compounds of the worm. For the data analysis, they used LabVIEW VI. The results showed the signature of the volatile compounds emitted by the worm. Thus, it was the first time the eNose system detected the fall armyworm.

Among the studied papers, none have been found that compares a large group of ML techniques for determining adulteration and characterising essential oils. The most similar cases were applied to evaluate the ripeness of bananas. The existing similar studies on essential oils consist of using mainly statistical methods and NN [20], using only NN [21], or using SVM [14]. Finally, none of the surveyed papers includes the eNose as part of a sensor network, which includes local resources such as a database and remote cloud computing.

## 3. Test Bench

In this section, the test bench is described. First, the features of the used prototype are detailed. Subsequently, the selected oil samples are mentioned, and the storing conditions and their origin is outlined. In the third place, the measuring methodology is explained. Then, the details of data preprocessing and other aspects of user data are identified. Finally, the selected methods for data classification and used metrics are fully described.

### 3.1. Prototype Description

**Figure 1:**
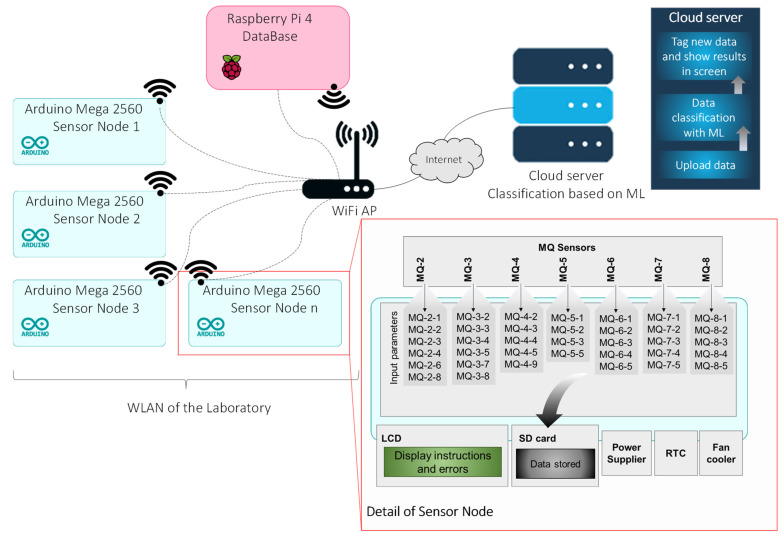
Proposed deployment of proposed gas sensor network in the laboratory, which includes ML in the cloud.

Regarding the used sensors, Figure 1 presents the gas sensor network proposal scheme for monitoring essential oils deployed in the laboratory. The network architecture consists of a series of sensor nodes composed of Arduino (Somerville, MA, USA) Mega 2560 microcontrollers with an MQ sensor array. In the wireless local area network (WLAN), a Raspberry Pi 4, which actuates as a database, was found. This database was used to avoid using the limited resources of the sensor nodes to store generated information. A Wi-Fi access point was used to connect all the wireless devices in the laboratory. Using the internet infrastructure of the laboratory, the network was remotely connected to a server in the cloud. In order to connect and put the sensor array into operation, the PuTTY 0.77 software was used. After the start of data storage in the DB, the WinSCP 5.21.7 software was used. This program allowed us to save the data in. csv for further processing. The sensor was configured to gather data every 1 min.

Regarding the array of sensors used, it was developed by Viciano-Tudela et al. [20]. A microprocessor, Arduino Mega 2560, was selected to establish the necessary functions of the sensor node. The array of sensors comprises seven sensors from the MQ family capable of detecting different gases (MQ-2, MQ-3, MQ-4, MQ-5, MQ-6, MQ-7, and MQ-8). Moreover, each sensor can present different responses for different gaseous compounds, having a total of 36 measured parameters. The measured parameters are the different parameters that each MQ sensor can measure; more details can be found in [13]. A total of nine different compounds can be detected, named in this paper as compounds 1 to 9. On average, each MQ sensor can measure five compounds according to their commercial calibrations and provided equations. The sensor node contains a screen (LCD), which allows you to see the instructions and the errors that can occur in the system. An SD card for temporal data storage and a real-time clock were included as a backup system to provide fault tolerance in the case of the wireless connection being lost. Finally, a power supplier and a cooling fan complete the sensor node components. The sensor node was deployed in a measuring chamber, as described in [20]. There were as many microcontrollers in the laboratory as there were required measuring chambers. Considering the low cost of the proposed system, its adaptability, and its scalability, many measuring chambers can be implemented in the same laboratory.

The information the MQ sensors detects was stored in a Raspberry Pi 4 that acts as a database. These were connected to an access point; in this way, the registered data could be accessed by using a server connected to the same access point through the available infrastructure of the building. Data classification was carried out in the server cloud using ML techniques. Finally, after classifying the data, the results and graphs could be visualised.

### 3.2. Oil Samples

A total of six different types of samples were used to carry out this study. On the one hand, the herbal essential oil of *Cistus ladanifer* and *Pinus pinaster* as well as the adulterated oil of *Cistus ladanifer* with *Pinus pinaster* were used. These oils’ origin and processing steps were described in a previous study by Viciano-Tudela et al. in 2023 [20].

**Figure 2:**
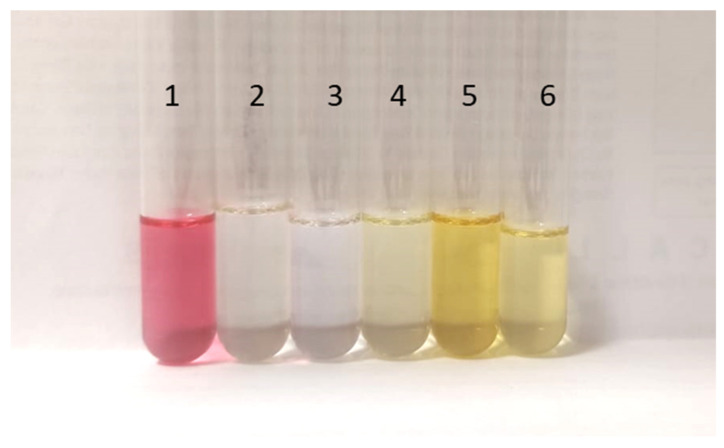
Essential oils used for the tests (1 red fruit; 2 *Melaleuca alternifolia*; 3 *Pinus pinaster*; 4 *Citrus Limonium*; 5 *Cistus ladanifer*; and 6 adulterated *Cistus ladanifer* with *Pinus pinaster*).

On the other hand, an additional herbal oil, the commercial essential oil of *Melaleuca alternifolia*, commonly known as tea tree (100% tea tree oil 18 mL from Drasanvi, reference: CP190523), was used. In addition, two essential oils from fruits were included to generate a more complex and variated database. The first fruit-based essential oil that was used was lemon oil (*Citrus limonium*). It is a commercial oil (lemon essential oil 12 mL from Labiatae, lot: 170870620). The second oil came from red fruits, and it was a mixture of commercial essential oils, among which red fruit aroma was added. It was a sample of commercial oils (Carrefour refill red fruit stick air freshener 250 mL). In Figure 2, images of the samples of essential oils used are shown.

To maintain their physicochemical characteristics, the samples were kept refrigerated at 5 °C until their processing in a translucent glass flask; this prevented light from affecting the compounds that make up the sample. In addition, they were kept entirely closed and hermetic inside another flask to avoid potential or accidental losses.

### 3.3. Measurement Methodology

The measurement chamber used and described by Viciano-Tudela et al. [20] in a previous sampling was used to obtain the data. The blank tests to analyse the sensors’ drift were already conducted and analysed in the same paper. In this case, 2 mL were used in a glass vial using an automatic pipettor. After preparing the samples, they were allowed to warm to room temperature after removing them from the refrigerator. In the meantime, we performed a measurement chamber blank. The gas sensor was turned on, and the measurement chamber was closed and empty. This allowed the burning of previous residues deposited on the sensing elements of the MQ sensors.

After making the blank, the previously tempered vial was introduced individually. In this case, the first hour of data was also removed. This allowed the volatile compounds of the essential oils to permeate and reach the volume of the measurement chamber.

**Figure 3:**
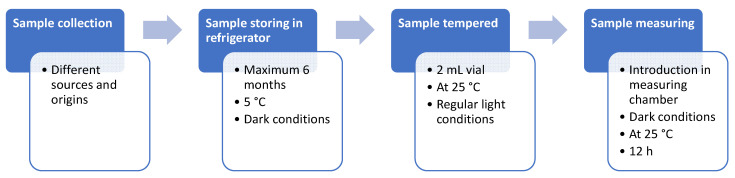
Diagram of the methodology for storing, tempering, and analysing essential oil samples.

In addition, the blank was previously repeated in each data collection of each of the oils. Therefore, the complete elimination of the volatile compounds of the previous oil was ensured, avoiding interference in the responses of the sensors. Below is a simplified diagram of the methodology used to obtain the data (Figure 3).

### 3.4. Data Preprocessing

First, all gathered data were filtered and normalised to 1 according to Equation (1). This Equation was applied for each measured parameter. Regarding filtering data, those parameters with data equal to 0 along all the tests were extracted from the dataset. This situation was only detected for MQ-3 sensor for the parameters MQ3–4, MQ-5, and MQ3–7.

(1) $$X_{normalised}={(X}_{i}-X_{min})/{(X}_{max}-X_{min})$$

where *X_i_* is the data to be normalised, *X_min_* is the minimum value of the parameter *X*, and *X_max_* is the maximum value of the parameter *X*.

In this paper, two different approaches regarding the input data were compared. On the one hand, as the first option, tests were conducted using all the gathered data, including all the individual measurements obtained during the sampling time. In this case, no additional data preprocessing was conducted to simulate real-time measurement scenarios. The number of measurements varies among the sources used. Table 1 summarises the number of measurements collected for each sample. For each measure, the data of each used sensor were gathered. It is essential to consider that each individual sensor provides data from two to five parameters corresponding to different chemical compounds. On the other hand, tests were performed using the mean values of the obtained data. In this case, the preprocessing included the calculation of the average value of all gathered data every hour for each sensor and compound. We designed the test bench to collect datasets of different sizes to evaluate their potential effect on the classification.

To reduce the data for the classification, and considering that previous results have already indicated that most of the sensed parameters are correlated with other ones, an initial selection of data was made. We aimed to reduce the number of included parameters in the classification algorithm to two. Thus, a multivariate analysis was performed, and according to the results, different data combinations were included as inputs for the classification algorithms. The combinations included (a) using parameters with a significant correlation, only one parameter per sensor; (b) using all parameters with a significant correlation; and (c) using all parameters. The multivariate analyses concluded that 15 parameters had a significant correlation. The parameters were defined by the name of the sensors and the monitored environmental compound (names from 1 to 9 in [20]. The parameters were MQ7–3, MQ7–1, MQ7–2, MQ8–1, MQ8–2, MQ7–4, MQ3–2, MQ6–2, MQ6–5, MQ8–5, MQ6–1, MQ8–3, MQ8–4, MQ3–3, and MQ6–4 in decreasing order of correlation. The parameters for option a) included using MQ7–3, MQ8–1, MQ3–2, and MQ6–2. In this particular case, we studied in detail using only the first two parameters or the first three parameters or all of them.

### 3.5. Classification

In this subsection, the classification algorithms and used software are detailed. Regarding the selected software, Matlab R2021 was used. Considering that the paper aimed not to perform a binary classification, the number of available algorithms was limited to DA, SVM, NB, NN, and KNN. The data included in the analyses comprised all the obtained datasets with or without pretreatment, according to the studied data, as individual datasets. The datasets were reduced in terms of included parameters according to the results of the multivariate analysis, using 2, 3, 4, 15, or 33 parameters. For all the algorithms, the same datasets were used, ensuring that there were no differences in the training process. The selected functions on Matlab for this classification included fitcdiscr, fitcnb, fitcecoc, fitcknn, and fitcnet. In all the cases, the used predictor variables were different combinations of sensed parameters included as a table, and the labels were the type of measured essential oil included as a cell.

A series of metrics were used to allow a fair comparison of results and to evaluate the performance of the different data preprocessing options. The metrics were based on the number of true positives (*TP*), false positives (*FP*), true negatives (*TN*), and false negatives (*FN*) from the obtained confusion matrixes after each classification. Among existing metrics, the selected ones based on literature [38] were precision, recall, F1-score, and accuracy, as shown in Equations (2)–(5):

(2) $$precision=\frac{TP}{TP+FP}$$

(3) $$recall=\frac{TP}{TP+FN}$$

(4) $$F1-Score=2\times \frac{precision\times recall}{precision+recall}$$

(5) $$accuracy=\frac{TP+TN}{TP+TN+FP+FN}$$

These metrics will allow a comparison of obtained *precision*, *recall*, *F*1*-score*, and *accuracy* among ML algorithms and tested data treatment options in the results and with proposals of other authors in the discussion. These are well-known metrics widely used in multiple studies.

**Figure 4:**
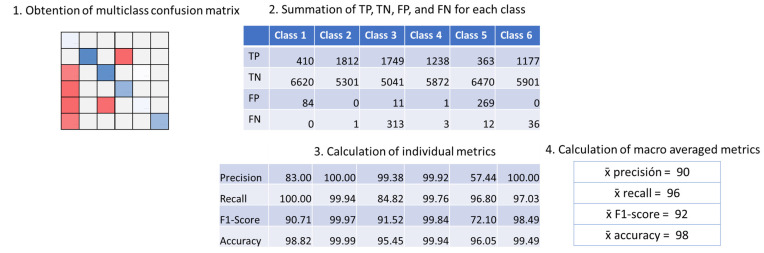
Steps followed for macro-averaged metric calculation.

The metrics were then calculated for every single class (*j_class_*), considering as: (i) *TP*, the number of cases correctly classified; (ii) *FP*, the number of cases classified as *j_class_* belonging to other classes; (iii) *FN*, the number of cases of *j_class_* classified as other classes; and (iv) *TN*, the number of cases from other classes classified as non-*j_class_*. Then, the mean of the obtained metric was calculated for the six classes to calculate the macro-averaged metric. The macro-averaged metrics are the ones presented in the paper. In order to facilitate comprehension, a summary of the following process can be seen in Figure 4.

## 4. Results

This section may be divided by subheadings. It should provide a concise and precise description of the experimental results, their interpretation, as well as the experimental conclusions that can be drawn.

Before analysing the performance of classification for the different algorithms in detail, Table 2 summarises some statistical information about gathered raw data. We can see that there is a huge variability among the obtained values. In this data, we analysed all gathered data without differentiation among oil origins.

### 4.1. Data Classification with All Measured Values

**Figure 5:**
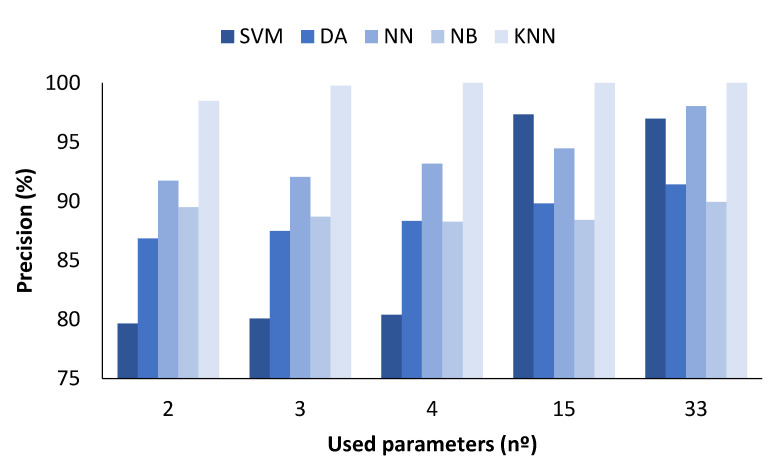
Comparison of attained precision values for different ML algorithms and number of used parameters when all measured values are used.

**Figure 6:**
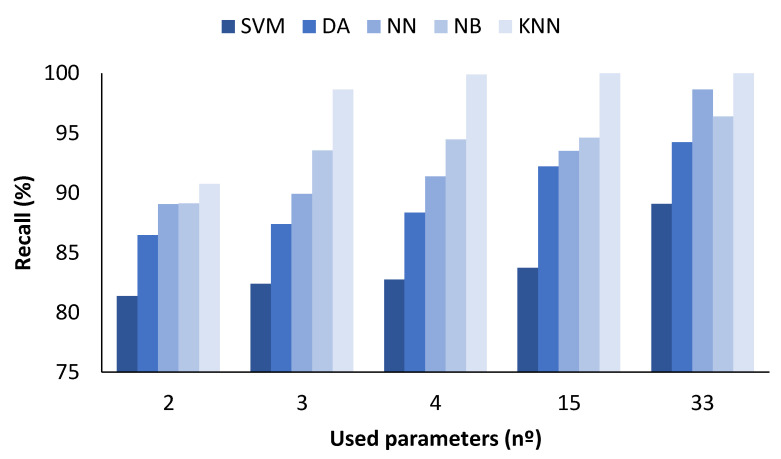
Comparison of attained recall values for different ML algorithms and number of used parameters when all measured values are used.

**Figure 7:**
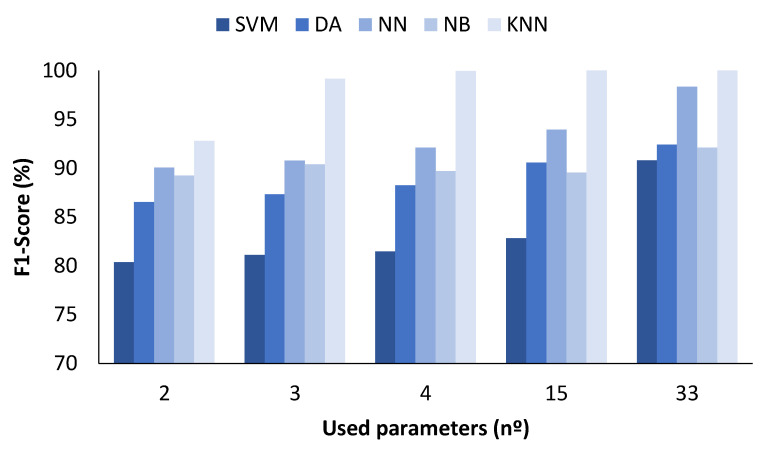
Comparison of attained F1-score values for different ML algorithms and number of used parameters when all measured values are used.

**Figure 8:**
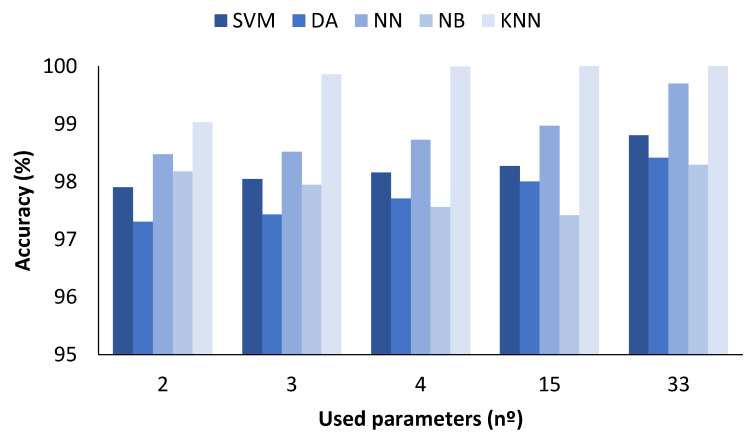
Comparison of attained accuracy values for different ML algorithms and number of used parameters when all measured values are used.

In the figures presented below (Figure 4, Figure 5, Figure 6 and Figure 7), the results obtained considering all the values are presented. The metrics represented are precision (%) (Figure 5), recall (%) (Figure 6), F1-score (%) (Figure 7), and accuracy (%) (Figure 8).

In Figure 5, the X-axis represents the number of parameters included in the classification algorithm, while the Y-axis indicates the attained macro-average precision. The results show the differences regarding the use of the different methods. The SVM method showed a higher precision when the number of parameters used was 15 or more, with a precision of 97.32 and 96.97%, respectively. For the DA method, the best results were also achieved for the number of parameters 15 or 33, with the value being 92.20 and 94.24%, respectively. Regarding the NN, a precision of 98.63% when the number of cases is 33 was attained. The best precision values for the NB ML algorithm were obtained when the number of used parameters were 2 and 33, with 89.49 and 89.95%, respectively. Finally, the method that showed the highest precision values for all cases was the KNN model. The precision was 90.75% for 2 parameters, 98.62% for 3 parameters, 99.90% for 4 parameters, and 100% for 15 or more parameters. Therefore, after analysing the precision results, we can conclude that the best model is the KNN model.

In Figure 6, the results for the different ML algorithms used are compared, and the results detailed below were obtained. First, the SVM had a lower recall, ranging from 81.36 to 89.08%, when the number of parameters used was 2 or 33, respectively. Concerning the DA, the recall was also higher when the number of parameters was 33, attaining 94.24%. The classification with the NN achieved a 98.63% recall when all parameters were used. For the NB algorithm, the recall was 96.39% when the 33 parameters were used as input information. Finally, for the KNN method, it presented a 100% recall for 15 or more parameters.

Figure 7 shows the results for the F1-score. The maximum F1-score for SVM ML algorithms was 90.80% and was achieved when the number of parameters was 33. Regarding the DA, the values are similar regardless of the number of parameters used, ranging from 86.54 to 92.44%. There is a clear difference regarding the NN algorithm when the number of parameters used was 33, which gave a value of 98.32% compared with the range from 90.08 to 93.94% when 2 to 15 parameters were used. The results with the NB are characterised by similar F1-score values independent of the number of parameters included, with maximum and minimum values of 89.26 and 92.10%, respectively. Finally, the KNN method was the one that attained the best performance. The F1-score was 92.80% for 2 parameters, 99.16% for 3, 99.94% for 4, and 100% for 15 and 33 parameters.

The accuracy results for the different numbers of parameters used can be seen in Figure 8. The ML algorithms used present differences in terms of attained accuracy values. The best accuracy for the SVM ML algorithm was achieved when the number of parameters used was 33. The obtained accuracy for this case was approximately 98.80%. Regarding the DA ML algorithm, similar accuracies were attained regardless of the number of used parameters, ranging from 97.30 to 98.4% for 2 and 33 parameters, respectively. Concerning the results when the applied classification algorithm was NN, the obtained accuracy reached 99.69% when 33 parameters were used. The accuracy values for NB presented barely any variation independently of the used parameters, ranging from 97.42 to 98.29% for 15 and 33 parameters. In this case, the accuracies for the 2 and 33 parameters were almost identical. Finally, with the KNN algorithm, the achieved accuracies were the best, with 100% accuracy for 15 and 33 parameters, 99.99% for 4 parameters, 99.85% for 3 parameters, and 99.02% for 2 parameters.

### 4.2. Data Classification with Calculated Mean Values

In this subsection, the obtained results, in terms of calculated metrics, are described when the hourly mean values are used for the classification. As in the previous subsection, we first analyse the precision, followed by the recall, F1-score, and accuracy results.

**Figure 9:**
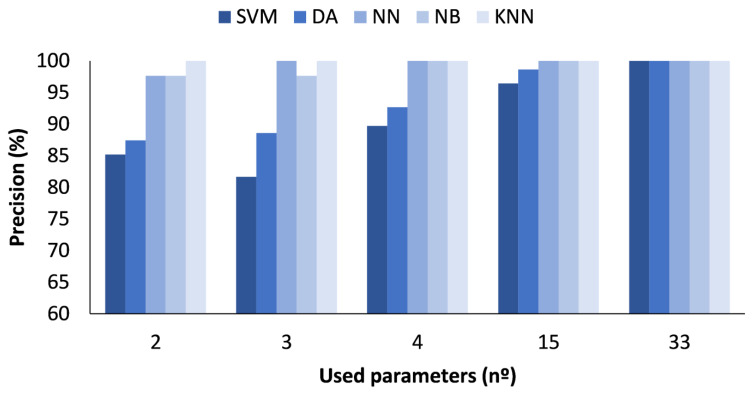
Comparison of attained precision values for different ML algorithms and number of used parameters when mean values are used.

Figure 9 displays the summary of macro-averaged precision for the different classifications. The obtained precisions ranged from 81.17 to 100%. The worst case corresponded to the classification performed with SVM using 3 parameters. With all employed ML algorithms, 100% precision was obtained when all parameters were used. Three ML methods attained 100% precision with fewer parameters, particularly NB with 4 and 15 parameters; KNN with 2, 3, 4, and 15 parameters; and NN with 3, 4, and 15 parameters. The precision within the case with the most limited number of parameters was 85.17% for SVM, 87.43% for DA, 97.62% for NB, 97.62% for NN, and 100% for KNN, respectively.

**Figure 10:**
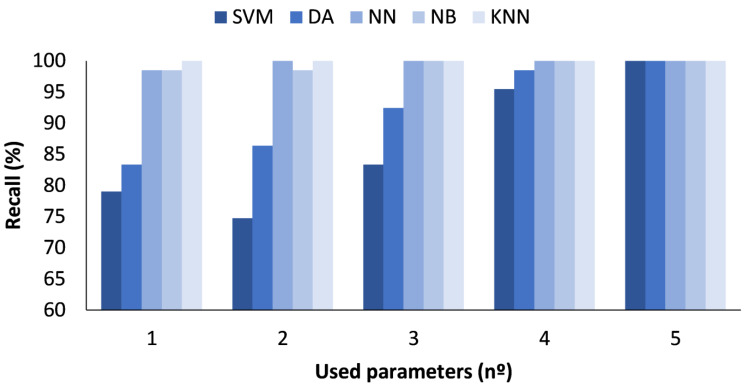
Comparison of attained recall values for different ML algorithms and number of used parameters when mean values are used.

The macro-averaged recall for the different classifications is displayed in Figure 10. The obtained precisions ranged from 100 to 74.75%. The lowest recall was achieved with the classification performed with SVM using 3 parameters. In all the cases, 100% recall was achieved when all parameters were used, regardless of the employed ML algorithm. The recall within the case with the most limited number of parameters was 79.04% for SVM, 83.33% for DA, 98.48% for NB, 98.48% for NN, and 100% for KNN. A 100% recall with fewer parameters was attained with the following ML algorithms: NB with 4 and 15 parameters; KNN with 2, 3, 4, and 15 parameters; and NN with 3, 4, and 15 parameters.

**Figure 11:**
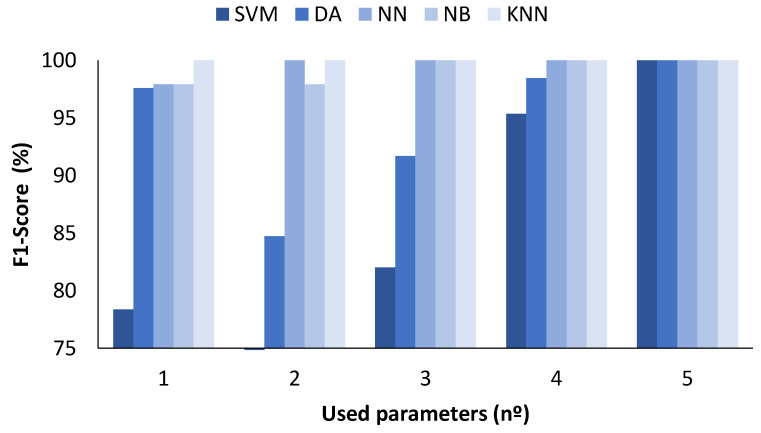
Comparison of attained F1-score values for different ML algorithms and number of used parameters when mean values are used.

Figure 11 shows the macro-averaged F1-score for different classifications, with the minimum and maximum F1-score obtained being 74.28% and 100%, respectively. The worst performance was found in the SVM classification with 3 parameters, whereas an F1-score of 100% was obtained when all parameters were used. The NB method with 4 and 15 parameters; KNN with 2, 3, 4, and 15 parameters; and NN with 3, 4, and 15 parameters could achieve an F1-score of 100% with fewer parameters. The most limited case, with several parameters equal to 2, had an F1-score of 78.40% for SVM, 97.60% for DA, 97.38% for NB, 97.92% for NN, and 100% for KNN.

**Figure 12:**
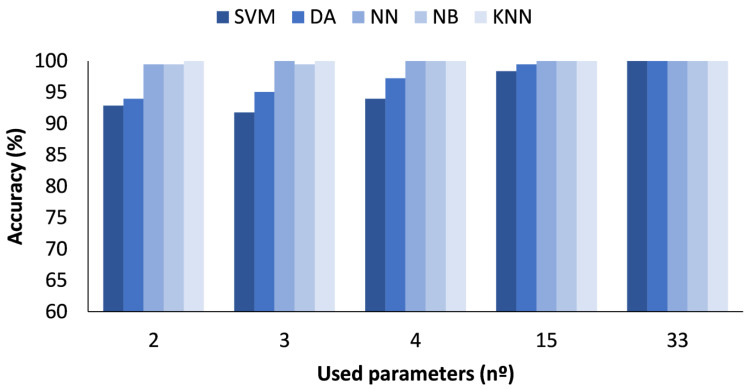
Comparison of attained accuracy values for different ML algorithms and number of used parameters when mean values are used.

The macro-averaged accuracy for the generated data classifications is outlined in Figure 12. The obtained accuracies ranged from 78.39 to 100%. The minimum accuracy value was obtained with the classification performed with SVM using 3 parameters. Except for SVM, in all the ML algorithms tested, an accuracy of 100% was achieved when all parameters were used. The accuracy in the case with the most limited number of parameters was 78.39% for SVM, 97.60% for DA, 97.92% for NB, 97.92% for NN, and 100% for KNN. A 100% accuracy with fewer parameters was attained with the following ML algorithms: DA with 15 parameters; NB with 4 and 15 parameters; KNN with 2, 3, 4, and 15 parameters; and NN with 3, 4, and 15 parameters.

## 5. Discussion

In this section, we discuss the obtained results in terms of the most suitable configuration for the proposed eNose for the classification of essential oils. First, we deal with the results of the different tested classification methods and input data. Subsequently, we compare our results with existing solutions based on eNoses for similar cases. Later, we describe the impact of the proposed research. Finally, the limitations of conducted tests and analysis are mentioned.

### 5.1. Discussion of Obtained Results and Selection of the Most Suitable Configuration

In this subsection, we discuss the obtained results beyond using the included metrics. The details of the main results are structured in the subsequent paragraphs describing the following aspects: (i) most common errors in performed classifications; (ii) selection of most suitable ML technique for classification; and (iii) evaluation of data preprocessing impact.

First, in most cases, a significant number of misclassifications were related to the essential oil from red fruits, which was classified in some cases as lemon. This misclassification was found in SVM, NB, and KNN, having 0 TP for the red fruits’ essential oil class in some cases. A possible explanation is that the red fruits dataset was one of the smallest datasets, and this might have an impact on the classification performance. The other general misclassification, mainly found in DA, was the misclassification of adulterated *Cistus ladanifer* essential oil as 100% *Cistus ladanifer* essential oil. This misclassification was mainly linked to the results when data were not merged hourly. In this case, the dataset of *Cistus ladanifer* was one of the largest datasets. Thus, the size of the datasets does not seem to be a determining factor once a minimum number of data points are collected (at least 400 data points).

Regarding the most suitable technique for data classification, according to the results shown in the previous section, we calculated macro-averaged metrics for each of the studied ML techniques (see Table 3). In this case, we calculated the metrics joining the results for the different number of included parameters, merging or not merging the data hourly. According to data from Table 3, the best ML technique for obtained metrics was KNN followed by NN. The worst results were obtained when SVM was used.

**Figure 13:**
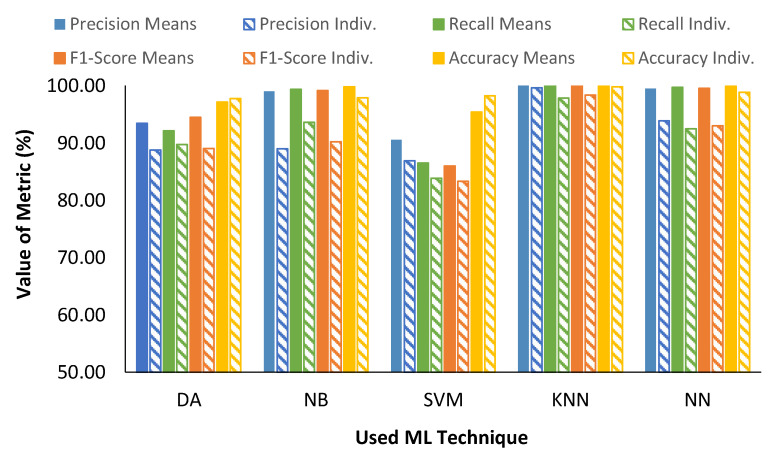
Summary of performance of different tested ML algorithms and data preprocessing.

The following aspect to be considered is the suitability of using individual data or the hourly mean data for classification. The macro-averaged metrics for each ML algorithm combining the different number of parameters divided into individuals (Indiv.) or mean data can be seen in Figure 13. In most cases, the use of hourly mean data implied the attainment of better performance in the studied metrics. Remarkably, the highest differences were for NB and NN, in which cases the use of individual data supposes a decrease in metrics performance of up to 5% compared with the metrics obtained with hourly data. A decrease of 3% in the metrics performance was found in DA when the individual data were used. The differences were minimum for SVM and KNN, the latter of which had a lower decrease and very similar precision and accuracy values. Finally, we should highlight the case of the DA and SVM, in which the use of individual data produced an increase in accuracy. Thus, we can affirm that in all the cases, the use of averaged hourly data is preferred. Nonetheless, individual data can be used considering the possible future requirements for fast classification. When individual data should be used, selecting the KNN algorithm to classify the samples is preferred.

### 5.2. Comparison with Existing eNoses for Similar Applications

In this subsection, we compare our results with the current relevant literature on using ML and eNoses for classifying food and oils (see Table 4). There are mainly two general applications of ML and eNoses in food and related products: the identification of adulterated products and the classification of products. For the detection of fraudulent products, the most common case is for the detection of adulteration in edible [28,39,40] and essential oils [20,21]. In some cases, it is also applied to meat [30]. Concerning the identification of products, which is the case with which we are dealing, there is a higher variability of products to which this has been applied. The found cases cover the ripening of bananas [33], the differentiation of fish and meat types and their quality [41], the growing conditions of potatoes [42], and the identification of different products such as edible oils, cheese, and wine [43,44]. As detailed before, there are a few cases in which ML and eNoses are applied to essential oil, and their application was to detect adulterated essential oils [20,21]. In our proposal, we identified the type of essential oil, with five types of oil, and we included one case of adulterated essential oil, having in total six samples.

Focusing on the classification methodologies, the papers used, on average, four different techniques. The maximum number of techniques was seven in [33,39], and the minimum was one in [42]. Nonetheless, some of the applied techniques were not ML or variations of the same ML technique used. Considering only the number of applied different techniques based on ML, the average was three, the maximum was six [39], and the minimum was one [20,21,42]. In this paper, five different techniques were used. The most used ML technique was ANN, which was used in six out of the eleven included papers [20,28,30,39,40,42]. This is followed by SVM [30,33,39,40,44] and KNN [33,39,40,43,44], which were utilised in five papers, and DA [28,41,44] and NB [33,39,40], which were each used in three papers. All these techniques, NN, SVM, KNN, DA, and NB, were included in this paper. AdaBoost (AB) was used in some examples [39,40]. The less used techniques were decision tree classifier (DTC), gradient boosting classifier (GBC), and classification and regression trees (CART), which were used in [30,33,39], respectively. Most of the paper focused on multiclass classification for the different sorts of classification problems, multiclass or binary. There are only three examples in which binary classification was used [21,33,42]. Nonetheless, in [21], binary results were combined with multiclass classification results.

The last studied parameter was the number of included metrics. The standardised metrics allow a fair comparison. In some cases, the sole metric was the percentage of correctly classified cases [20,39]. Nonetheless, with this simple metric, it is not possible to fairly compare the outputs. Unfortunately, none of the included papers offered a wide range of metrics. Accuracy was the most offered metric beyond the percentage of cases correctly classified. Notwithstanding, it must be considered that in some cases, it is not possible to confirm that the authors mix the terms accuracy and percentage of cases correctly classified. The accuracy was provided by eight papers, [21,29,30,33,41,42,43]), the precision was provided by only one [28], and the recall was provided by only one [44].

A summary of existing proposals and the proposed solutions in this paper can be seen in Table 4.

### 5.3. Relevance of Proposed Sensor for Essential Oil Characterisation

In rural areas, valorising natural resources as an alternative and new business model can help fix the population. Exploiting natural resources such as *Cistus ladanifer* to obtain biomaterials (for example, essential oil or oleoresin) is directly linked to developing the specialised industry for product obtention, valorisation, and commercialisation. These products can be commercialised under local and sustainable seals. Nevertheless, as mentioned before, essential oils are susceptible to adulteration and must perform exhaustive monitoring of their quality and purity. The current methods for evaluating the source of essential oils are based on complex methods which cannot be easily applied. Thus, the proposed sensors are essential in ensuring the origin of local and sustainable products.

In the framework of the application of eNoses for *Cistus ladanifer* monitorisation, the developed sensor has different applications. First, the proposed sensor will be applied to monitorisation wild plants in a controlled environment to evaluate the variation of their scent in order to assess the content of oleoresin and essential oil of monitored individuals. Next, the sensor will be adapted to be able to measure the essence of the wild population of *Cistus ladanifer* in large groups of individuals to evaluate the optimal moment for harvest. In these two cases, the sensor will be implemented in a large wireless sensor network (WSN). In the first case, these WSNs will be composed of a significant number of nodes based on the MQ sensors. The WSN will become more complex in the second case, including soil and climatic sensors and remote sensing for plant phenotyping [45]. Finally, we will assess the suitability of this sensor for evaluating the quality of obtained essential oils, aiming to characterise the chemical compounds present in the samples or at least quantify the presence of main volatile organic compounds.

### 5.4. Limitations of Performed Tests

In this paper, we have proposed using alternative ML methods for determining the source of different essential oils. The system is based on data acquired in a series of measurements performed in laboratory conditions using a measuring chamber. The main limitation of the proposed system compared with existing solutions for the identification of essential oils are the following: (i) lower sensitivity than current laboratory equipment; (ii) impossibility of quantification and recognition of chemical products; or (iii) confusion in oils that have similar compounds. The main advantages of eNoses compared with traditional methods are (i) faster determination (in some cases); (ii) the limited or null use of reagents; (iii) no need for expert personnel to process the samples; and (iv) no destruction of analysed samples, allowing repetitions if necessary.

Regarding the proposed solutions, we highlight the inclusion of sensor nodes in a WSN to automatise the measuring process using measuring chambers. The main limitation of the proposed measuring chambers coupled with ML algorithms are the following: (i) they require at least 1 h of data to ensure high classification success; (ii) dependence on cloud computing for final classification; (iii) need to aerate the gas chamber between samples so as not to alter the data of the next oil tested; and (iv) wireless AP required to communicate the devices. Despite these limitations, the proposed system implemented measures to prevent the abovementioned risks. The local storing of data in the node and in the local database allows all data recovery in case the access point fails. Concerning the ML running on cloud computing, data classification can be conducted at another moment if accessing the cloud is impossible. This might not be a problem since the data classification in real-time is unnecessary.

Finally, the following issues must be considered concerning the proposed ML algorithms. The proposed data gathering, processing, and classification methodology have been designed for the tested essential oils. We only included six essential oils from five sources and one adulterated product. Considering the wide variety of essential oils used in different industries, including all the used products, it is impossible. We focused on herbal essential oils, such as *Cistus ladanifer*, *Pinus pinaster*, and *Melaleuca alternifolia*, including adulterated products, and we added two essential oils from fruits. Therefore, it should be considered that using this eNose in new products will require the generation of labelled information from this new source. Since ML algorithms can be adapted to new data, adding new essential oils is expected to endow the system with enhanced capabilities.

## 6. Conclusions

In this paper, we presented the application of an eNose based on MQ sensors integrated into a WSN with cloud computing for the correct characterisation of samples of six essential oils and adulterated products. Different algorithms were compared: DA, SVM, NN, BN, and KNN. The results indicated that using KNN can produce an accuracy, recall, F1-score, and precision of 1, 0.99, 0.99, and 1, respectively. Results improved when the input was the hourly average of collected data and when the number of included parameters increased. An accuracy of 100% was achieved with KNN for using 2 parameters (averaged data) or 15 parameters (individual data).

The main advantages of this system are its low cost, low maintenance requirements, easy use, and online operation. On the contrary, the main drawbacks are the limited number of analysed samples at the current moment, the possible misclassification of similar essential oils, and the necessity of aerating the chamber between samples.

Future work is linked to monitoring the network requirements to design the final network topology. In addition, integrating new sensors in the measurement chamber, such as optical or electromagnetic sensors, is being considered. Regarding the future application of eNoses in the natural environment, the requirements for deploying these networks and the most appropriate location of the sensors will be studied according to [46]. Other ML techniques, such as CART, RF, and GBC, will be studied, and larger data sets will be collected. Finally, once the prototype has been enhanced with the aforementioned capabilities, the results will be compared with commercial eNoses.

## Figures and Tables

**Table 1:** Summary of samples and the number of individual measurements.

Source	Acronym	Nº of Individual Measurements
*Cistus ladanifer*	Cla	1813
*Pinus pinaster*	Pp	1213
*Cistus ladanifer* + *Pinus pinaster*	CP	1241
*Melaleuca alternifolia*	Ma	410
*Citrus limonum*	Cli	2062
Red fruits	Rf	375

**Table 2:** General summary of obtained raw data.

Parameter	Maximum Value	Minimum Value	Mean Value	Standard Deviation
MQ2–1	116,856.0	0.0	434.8	3010.0
MQ2–2	933.0	31.0	160.1	146.8
MQ2–3	11,741.0	1.0	1164.1	1693.3
MQ2–4	2591.0	1.0	326.8	384.8
MQ2–6	1157.0	41.0	210.2	186.0
MQ3–2	266.0	0.0	9.3	13.1
MQ3–3	788.0	0.0	8.8	10.8
MQ3–8	36,118.0	0.0	15.0	441.0
MQ4–2	153,780.0	24.0	1308.9	2059.9
MQ4–3	878,916.0	0.0	763.0	11,344.1
MQ4–4	85,719.0	0.0	456.9	1602.7
MQ4–5	3012.0	79.0	607.2	422.1
MQ4–9	100,708.0	0.0	1861.9	3164.3
MQ5–1	332.0	19.0	96.8	56.0
MQ5–2	1183.0	32.0	160.4	148.0
MQ5–3	7033.0	81.0	1223.1	1153.1
MQ5–4	20,971.0	1.0	1220.1	1014.9
MQ5–5	356.0	59.0	155.8	56.4
MQ6–1	34,088.0	664.0	2540.4	1659.9
MQ6–2	2811.0	212.0	475.0	173.1
MQ6–3	153,519,925.0	44.0	92,054.9	3,167,065.0
MQ6–4	2,501,354.0	1.0	7710.1	35,559.1
MQ6–5	3939.0	288.0	670.6	265.1
MQ7–1	280.0	14.0	94.3	34.3
MQ7–2	903.0	0.0	13.3	33.0
MQ7–3	297.0	51.0	87.7	37.1
MQ7–4	1,891,349.0	1.0	3331.7	27,593.2
MQ7–5	25,483,977.0	1.0	4139.3	302,144.1
MQ8–1	10,450.0	3298.0	6410.1	1894.5
MQ8–2	7005.0	17.0	1554.7	1023.7
MQ8–3	7,913,118.0	1.0	232,761.3	293,845.6
MQ8–4	6031.0	134.0	2269.8	970.0
MQ8–5	70,742.0	0.0	3622.5	3862.6

**Table 3:** Macro averaged metrics joining the results for the different number of included parameters and both merging or not merging the data hourly.

ML Technique	Precision	Recall	F1-Score	Accuracy
DA	0.91	0.91	0.82	0.97
NB	0.94	0.97	0.95	0.99
SVM	0.89	0.85	0.85	0.97
KNN	1.00	0.99	0.99	1.00
NN	0.97	0.96	0.96	0.99

**Table 4:** Comparison of presented results with similar uses of eNoses in the food industry.

Year	Nº of Tech.	Used ML	Type	Applications	Products	Nº of Sensors	For	Accuracy	Precision	Recall	F1-Score	Ref.
2020	5	ANN and LDA	Multiclass	Adulterated products	Edible oils	8	ANN	0.9893	0.975	-	-	[28]
							*LDA*	0.942	0.897	-	-	
2021	3	ANN	Multiclass	Adulterated products	Essential oils	9	Mult.	0.989	-	-	-	[21]
			Binary				Bin.	1	-	-	-	
2021	7	GBC, SVM, NB, KNN, NN, and AB	Multiclass	Adulterated products	Olive oil	8	GBC	-	-	-	-	[39]
							SVM	-	-	-	-	
							NB	-	-	-	-	
							KNN	-	-	-	-	
							ANN	-	-	-	-	
							AB	-	-	-	-	
2022	4	SVM, DTC, and NN	Multiclass	Adulterated products	Beef and pork meat	9	SVM	0.8742	-	-	-	[30]
							DTC	0.8541	-	-	-	
							ANN	0.8885	-	-	-	
2023	2	ANN	Multiclass	Adulterated products	Essential oil	7	ANN	-	-	-	-	[20]
2023	6	ANN, SVM, KNN, NB, and AB	Multiclass	Adulterated products	Olive oil	8	ANN	0.6752	-	-	-	[40]
							SVM	0.8652	-	-	-	
							KNN	0.8989	-	-	-	
							BN	0.8202	-	-	-	
							AB	0.3483	-	-	-	
2019	2	KNN and f-RF	Multiclass	Identify products	General food	16	KNN	0.69	-	-	-	[43]
							RF	0.78	-	-	-	
2019	7	KNN, CART, NB, SVM, LSVM, and RF	Binary	Identify products	Banana	10	KNN	0.72	-	-	-	[33]
							CART	0.654	-	-	-	
							NB	0.602	-	-	-	
							SVM	0.494	-	-	-	
							LSVM	0.628	-	-	-	
							RF	0.718	-	-	-	
2021	4	QDA, QSVM, CSVM, and KNN	Multiclass	Identify products	Edible oils	4	QDA	0.589	-	0.69	-	[44]
							QSVM	0.953	-	0.966	-	
							CSVM	0.802	-	0.851	-	
							KNN	0.811	-	0.858	-	
2022	3	DA and RF	Multiclass	Identify products	Fish and meat	8	RF	0.95	-	-	-	[41]
							DA	-	-	-	-	
2023	1	KNN	Binary	Identify products	Potatoes	5	KNN	0.90	-	-	-	[42]
2023	5	DA, SVM, BN, KNN, and NN	Multiclass	Identify product	Essential oils	7	DA	0.9112	0.9092	0.4105	97.46	Proposed
							NB	0.9401	0.9651	0.4734	98.83	
							SVM	0.8874	0.8519	0.4234	96.82	
							KNN	0.9982	0.9893	0.4960	99.89	
							NN	0.9671	0.9609	0.4816	99.38	

## Data Availability

The data presented in this study are available on request from the corresponding author. The data are not publicly available due to privacy constraints.
